# Sinner or Saint?: Nck Adaptor Proteins in Vascular Biology

**DOI:** 10.3389/fcell.2021.688388

**Published:** 2021-05-26

**Authors:** Mabruka Alfaidi, Matthew L. Scott, Anthony Wayne Orr

**Affiliations:** ^1^Department of Pathology and Translational Pathobiology, Louisiana State University Health – Shreveport, Shreveport, LA, United States; ^2^Department of Cell Biology and Anatomy, LSU Health – Shreveport, Shreveport, LA, United States; ^3^Department of Molecular & Cellular Physiology, LSU Health – Shreveport, Shreveport, LA, United States

**Keywords:** actin cytoskeleton, receptor tyrosine kinase, SH2/SH3 domains, developmental biology, vascular biology, Nck1/Nck2, adaptor proteins, binding parners

## Abstract

The Nck family of modular adaptor proteins, including Nck1 and Nck2, link phosphotyrosine signaling to changes in cytoskeletal dynamics and gene expression that critically modulate cellular phenotype. The Nck SH2 domain interacts with phosphotyrosine at dynamic signaling hubs, such as activated growth factor receptors and sites of cell adhesion. The Nck SH3 domains interact with signaling effectors containing proline-rich regions that mediate their activation by upstream kinases. In vascular biology, Nck1 and Nck2 play redundant roles in vascular development and postnatal angiogenesis. However, recent studies suggest that Nck1 and Nck2 differentially regulate cell phenotype in the adult vasculature. Domain-specific interactions likely mediate these isoform-selective effects, and these isolated domains may serve as therapeutic targets to limit specific protein-protein interactions. In this review, we highlight the function of the Nck adaptor proteins, the known differences in domain-selective interactions, and discuss the role of individual Nck isoforms in vascular remodeling and function.

## Adaptor Proteins in Signal Transduction Research

Mammalian cells express more than 500 kinases and more than 200 phosphatases that regulate nearly every aspect of cellular function. Regulated spatial organization enhances the specificity of these kinase and phosphatase reactions to generate specific cellular outputs. To accomplish this organization, the cell utilizes a host of modular adaptor proteins to mediate specific protein-protein interactions through various interacting domains (Birge et al., 1996). While lacking enzymatic activity, these modular adaptor proteins utilize various interacting domains to regulate a variety of cellular processes, and their deletion often results in embryonic lethality (Bladt et al., 2003). This review focuses on the non-catalytic region of tyrosine kinase (Nck) family of adaptor proteins that couples tyrosine phosphorylation to changes in cell morphology, gene expression, and function in a variety of vascular diseases. In it, we discuss the classic role of Nck adaptor proteins in cytoskeletal remodeling and vascular morphogenesis and new roles for Nck isoforms in the regulation of gene expression and vascular cell phenotype.

## Nck Family of Signaling Adaptors

The Nck family of Src homology 2 (SH2) and SH3 domain-containing adaptor proteins, including Nck1 (Nckα) and Nck2 (Nckβ, Grb4) were first identified in 1990 (Lehmann et al., 1990) and were subsequently isolated from multiple organs, including the brain, heart, lung, liver, and kidney (Li et al., 1992; Park and Rhee, 1992). The Nck1 gene localizes to chromosome 3q21 in a region mutated in various cancers (Chen et al., 1998), and genome-wide association studies (GWAS) link Nck1 single nucleotide polymorphisms (SNPs) with obesity, dyslipidemia, circulating leukocyte counts, platelet counts, and coronary artery disease (Locke et al., 2015; van der Harst and Verweij, 2018; Chen M. H. et al., 2020). The Nck2 gene localizes to chromosome 2 band q12 (2q12) in a region linked to neurological development (Chen et al., 1998; Griggs et al., 2009; Hladilkova et al., 2015), and GWAS analysis link Nck2 SNPs to drug addiction, Alzheimer’s Disease, and platelet levels (Liu et al., 2013; Chen M. H. et al., 2020; Schwartzentruber et al., 2021). While Nck1 shows similar expression across adult tissues, Nck2 shows higher expression in the spleen and skeletal muscle and lower expression in the liver (Chen et al., 1998; Bladt et al., 2003; Latreille et al., 2011). In addition, several studies have shown altered Nck1/2 expression in disease states. Multiple cancer models show enhanced Nck1 expression, driven at least in part by the expression of the Nck1-antisense (AS) LncRNA, which competes for miR-137 binding to Nck1 mRNA (Deshpande et al., 2019; Chang et al., 2020; Liu et al., 2020). Similarly, metastatic melanoma cell lines shower higher Nck2 expression compared to non-metastatic melanoma (de Wit et al., 2005; Labelle-Cote et al., 2011). In addition, cardiometabolic disease is also associated with elevated Nck1 expression, as Nck1 expression is enhanced in endothelial cells overlying human atherosclerotic plaques compared to healthy vessels and in adipocytes in white adipose tissue from obese or aging mice (Haider et al., 2018; Alfaidi et al., 2020a). However, Nck2 shows reduced expression in white adipose tissue from obese humans (Dusseault et al., 2016), suggesting Nck1 and Nck2 may play differential roles in cardiometabolic disease. While differential expression may be due to altered transcription, Nck1 and Nck2 also show differential regulation by post-translational degradation. In podocytes, c-Cbl mediates the ubiquitination of Nck1, but not Nck2, on Lys178 resulting in selective Nck1 degradation (Buvall et al., 2013).

**FIGURE 1:**
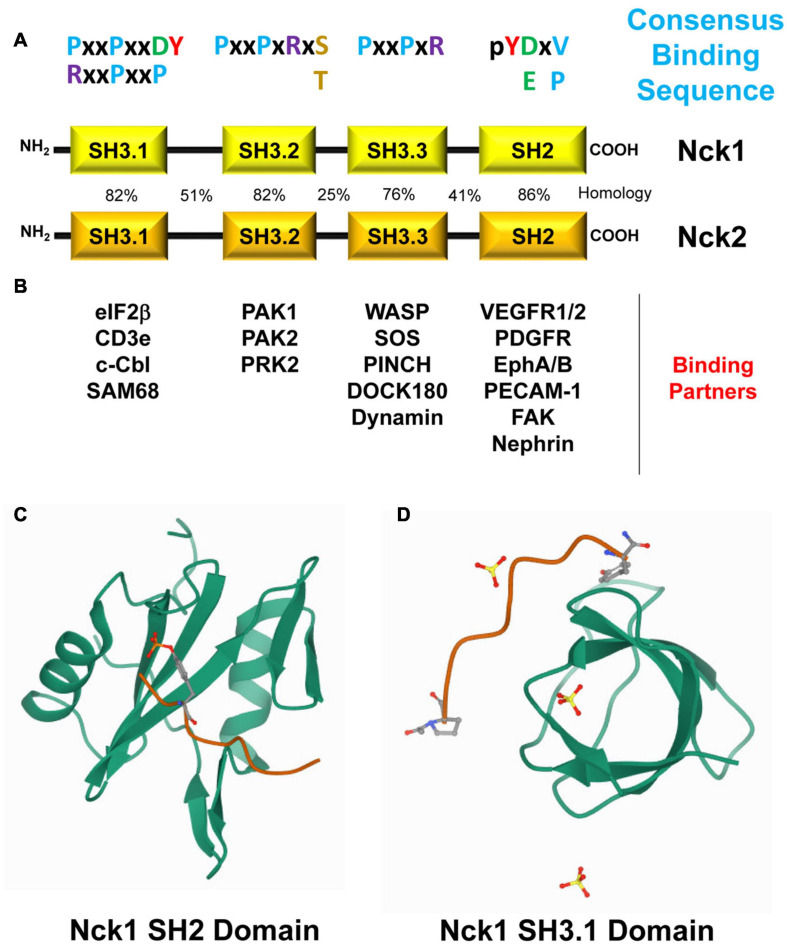
**(A)** Modular Structure of Nck1 and Nck2 and their binding partners. Nck1 and Nck2 consist of one C-terminal Src-homology domain 2 (SH2) and three N-terminal SH3 domains. The sequence homology in humans (%) of each domain, their consensus binding sequence, and their known binding partners are shown. **(B)** Selected Nck binding partners and the domains of Nck that mediate these interactions are shown. **(C)** The Nck1 SH2 domain (PBD ID: 2CI9, RCSB Protein Data Bank, https://www.rcsb.org/) complexed with a phosphotyrosine peptide (Frese et al., 2006). **(D)** The Nck1 SH3.1 domain (PBD ID: 5QU2, RCSB Protein Data Bank, https://www.rcsb.org/) complexed with a PPPVPNPDY peptide (Richter et al., unpublished).

Despite being expressed by different genes, Nck1 and Nck2 in humans share 68% amino acid identity and 79% similarity (Figure 1A) and possess both conserved and distinct downstream signaling partners (Chen et al., 1998; Lettau et al., 2009; Figure 1B). The C-terminal SH2 domain in Nck binds to specific phosphotyrosine residues (pTyr) at dynamic signaling hubs, such as activated growth factor receptors and sites of cell adhesion (Becker et al., 2000). Through its three tandem SH3 domains, Nck interacts with proteins containing proline-rich regions to form a local signaling scaffold to recruit proteins involved in cytoskeletal remodeling and regulation of gene expression (Pawson, 1995; McCarty, 1998; Lettau et al., 2009; Chaki and Rivera, 2013).

## Nck 1/2 Domains and Interacting Proteins

The modular architecture of adaptor proteins allows for numerous individual and potentially simultaneous protein-protein interactions (McCarty, 1998). Over the past decade, more than 60 Nck interaction partners have been identified, mostly involved in actin cytoskeleton organization (Bokoch et al., 1996; Galisteo et al., 1996; Lu et al., 1997; Bokoch, 2003). While many Nck1/2 binding partners interact with both isoforms, some interactions preferentially occur with select Nck isoforms and may therefore confer isoform-selective functions (Lettau et al., 2009, 2010; Jacquet et al., 2018). In this section, we discuss the structure, function, specificity, and regulation of the SH2 and SH3 domains that make up the Nck family adaptor proteins.

### SH2 Domains

Phosphotyrosine promotes signaling complex formation by recruiting signaling proteins with phosphotyrosine-binding (PTB) domains, such as SH2 domains and PTB domains. SH2 domains consist of ∼100 amino acids forming a central anti-parallel β-sheet and two flanking α-helices that bind short phosphotyrosine motifs in interacting proteins (Figure 1C; Waksman et al., 1992; Liu B. A. et al., 2006). A positively charged pocket with a conserved Arg residue on one side of the central β-sheet contributes to high-affinity phosphotyrosine binding, whereas an extended binding surface on the other side contains more variable residues that contribute to domain-selective interactions with sequences C-terminal to phosphotyrosine (Waksman et al., 1992). The human and mouse genome encodes for 120 SH2 domains across 110 different proteins that regulate protein interaction (adaptors, scaffolds), phosphorylation, small GTPase activation, cytoskeletal and chromatin remodeling, signal regulation, and protein ubiquitination (Liu B. A. et al., 2006).

The Nck1 and Nck2 SH2 domains show both similarities and differences in their binding properties. The Nck1 and Nck2 SH2 domains bind to a similar pY-D/E-x-V/P consensus sequence in target proteins (Figure 1A), although alterations in the C-terminal amino acids may affect binding specificity and affinity (Frese et al., 2006). Phosphorylation of Platelet Endothelial Cell Adhesion Molecule-1 (PECAM-1) on Tyr663 (pYTEV) supports binding to both the Nck1 and Nck2 SH2 domain (Machida et al., 2007; Alfaidi et al., 2020b), whereas phosphorylation on the similar Tyr686 (pYSEV) site did not support binding of either Nck1 or Nck2 (Machida et al., 2007). Moreover, both Nck1 SH2 and Nck2 SH2 bind to platelet derived growth factor (PDGF) and epidermal growth factor (EGF) receptors, but the Nck2 SH2 domain binds with a higher affinity than the Nck1 SH2 domain (Chen et al., 1998). This differential affinity may be due to Nck1 SH2 domains and Nck2 SH2 domains binding at different sites, as the PDGF receptor phosphotyrosine residue pY751 specifically interacts with Nck1 (Nishimura et al., 1993), whereas pY1009 specifically interacts with Nck2 (Chen et al., 2000). Similarly, phosphorylated ephrinB1 associates specifically with the Nck2 SH2 domain but not with the Nck1 SH2 domain (Stein et al., 1998; Pesti et al., 2012). Additional information on Nck1/2 SH2 identified binding partners and their interacting sequences are provided in Table 1.

**TABLE 1:** Some of the known Nck1/2 SH2 binding partners.

Binding partner	Binding site	Nck isoform	pTyr sequence	Reference
PDGFRB	PY751	Nck1	VDpYVPMLDMK	Nishimura et al., 1993
EPHB1	PY594	Nck1	KIpYIDPFTYE	Becker et al., 2000
PDGFR	PY1009	Nck2	VLpYTAVQPNE	Chen et al., 2000
FAK	pY397	Nck2	DDpYAEIIDEE	Goicoechea et al., 2002
VEGFR2	pY1214	Nck1/2?	FHpYDNTAGIS	Lamalice et al., 2006
Flt-1 (VEGFR1)	pY1333	Nck1/2?	VLpYSTPPI	Ito et al., 1998
Flt-1 (VEGFR1)	pY1213	Nck1/2?	VRpYVNAFKFM	Igarashi et al., 1998
Nephrin	pY1193	Nck1/2?	PLpYDEVQMGP	Verma et al., 2006
Cortactin	pY421	Nck1/2?	pYEDAASFKAE	Oser et al., 2009
PECAM-1	pY663	Nck1/2	VQpYTEVQVSS	Machida et al., 2007; Alfaidi et al., 2020b
BCR-Abl	pY177	Nck2, not tested for Nck1	PFpYVNVEFHH	Coutinho et al., 2000

*PY, phosphotyrosine; PDGFR, platelet derived growth factor receptor; VEGFR, vascular endothelial growth factor receptor; PECAM-1, platelet endothelial adhesion molecule 1; BCR, B-cell receptor; FGFR, fibroblast growth factor receptor; FAK, focal adhesion kinase.*

### SH3 Domains

Proline-rich regions in proteins bind to a variety of domains, such as SH3 domains, WW domains, and EVH domains. SH3 domains represent one of the largest families of modular domains, with humans expressing over 300 SH3 domains in over 200 proteins (Mayer and Gupta, 1998; Teyra et al., 2017). Composed of ∼60 amino acids, SH3 domains consist of five or six β-sheets arranged in two tightly packed anti-parallel β sheets forming a β-barrel (Figure 1D; Musacchio et al., 1992). The hydrophobic binding pocket of SH3 domains is enriched in aromatic and carboxylic amino acids with a conserved WPY triad critical for binding to proline-rich peptides (Musacchio et al., 1992; Chakrabarti and Janin, 2002). Mutation of the conserved Trp (W) residues in the WPY triad often results in significantly reduced binding efficiency (Lim and Richards, 1994; Lee et al., 1995). The hydrophobic binding pocket interacts with PxxP motifs in target proteins, while an adjacent RT loop and n-Src loop interacts with positively charged Arg/Lys residues on either side of the PxxP motif (Teyra et al., 2017). Proline-rich sequences can be divided into Class I motifs (RxxPxxP) and Class II motifs (PxxPxR) depending upon the relative position of the charged amino acids. However, several SH3 domains can bind peptides outside of this basic structure, such as R/K-rich peptides, thereby conferring greater specificity to the SH3 domain interactions (Teyra et al., 2017). While the SH3 does not show the same requirement for phosphorylation as observed for SH2 domains, phosphorylation of Ser and Thr residues adjacent to the conserved Arg/Lys residues (PxxPxRS/T) often results in reduced binding affinity (Howe and Juliano, 2000; Yurdagul Chen et al., 2013).

Several proteins interact with one or more of the Nck SH3 domains (Table 2; Pawson, 1995; Bokoch et al., 1996; Galisteo et al., 1996; Wang et al., 2000; Lettau et al., 2010). However, most interactions have not been tested for Nck1/2 selectivity (Lettau et al., 2010). Nck has three N-terminal SH3 domains, often referred to as SH3.1, SH3.2, and SH3.3. The Nck1 and Nck2 SH3.1 domain shows specificity for both class I and class II proline-rich sequences, with enhanced affinity to the class II sequence containing the PxxPxxDY motif (Aitio et al., 2008; Saksela and Permi, 2012). Phosphorylation of this Tyr residue abolishes ligand-binding affinity (Kesti et al., 2007; Takeuchi et al., 2008). The SH3.2 and SH3.3 domains exhibit high affinity for PxxPxR motif, with the second SH2 domain preferring a PxxPxRxS/T motif such as that found in the classic Nck-binding partner p21 activated kinase (PAK; Liu J. et al., 2006). The group I PAKs (PAK1/2/3) function as downstream effectors of the small GTPases Cdc42 and Rac-1. PAK normally exists as a transinhibited dimer, with an autoinhibitory domain of one PAK molecule binding and inhibiting the kinase domain of a second PAK. PAK activation involves its recruitment to phosphotyrosine-based signaling hubs in the plasma membrane through interaction with modular adaptor proteins, such as Nck and Grb2 (Li et al., 1992, 2001). PAK, through its conserved proline-rich region, binds to Nck SH3.2 and to a lesser extent to Nck SH3.3 (Bokoch et al., 1996; Galisteo et al., 1996; Lu et al., 1997; Bokoch, 2003). Upon membrane targeting, PAK can interact with active, GTP-bound Rac and cdc42 to relieve autoinhibition and promote PAK kinase activity. Active PAK contributes to cytoskeleton remodeling, actomyosin contractility, and activation of signaling pathways that regulate gene transcription (Manser et al., 1997; Bokoch, 2003).

**TABLE 2:** Some of the known Nck1/2 SH3 binding partners.

Binding partner	SH3 domain involved	Nck isoform	Proline rich sequence	Reference
PAK	SH3.2; less SH3.3	Nck1	KPPAPPMRNTSTM	Bokoch et al., 1996; Galisteo et al., 1996; Bokoch, 2003
WASP	SH3.1, SH3.2, SH3.3	Nck1	GRSGPXPPXP	Rivero-Lezcano et al., 1995
CYFIP2	SH3.1	Nck2	Uncharacterized	Lettau et al., 2010
FasL	SH3.2, less SH3.3	Nck2	PPLPLPPLKKR	Lettau et al., 2006
eIF beta	SH3.1, SH3.3	Nck1/2?	Uncharacterized	Kebache et al., 2002
Cortactin	SH3?	Nck1/2?	KPPVPPKP	Kruchten et al., 2008
CD3ε	SH3.1	Nck1/2?	NKERPPPVPNPDY	Sun et al., 2011
R-Ras	SH3.2; less SH3.3	Nck1/2?	PPSPPSAPRKK	Wang et al., 2000
SAM68	SH3.1	Nck1/2	Uncharacterized	Lawe et al., 1997
NIK	SH3	Nck2, not tested for Nck1	SODPCPPSRS/PRVPVRTTSR	Su et al., 1997

*PAK, P21-activated kinase; WASP, Wiskott-Aldrich syndrome protein; CYFIP2, cytoplasmic FMR1-interacting protein 2; FasL, Fas ligand; TCR, T cell receptor; FAK, focal adhesion kinase; SAM68, SRC associated in mitosis of 68 kDa; NIK, Nck inducing kinase; SAPK, stress activated protein kinase; eIF, eukaryotic initiation factor.*

Due to its inducible nature, Nck1/2 is generally recruited to signaling hubs through interaction between its SH2 domain and phosphotyrosine. However, this isn’t always the case. Nck’s SH3.1 domain can interact with PxxDY motifs on target proteins, such as the T cell receptor (TCR), to initiate signaling complex formation (Gil et al., 2002; Lettau et al., 2010). Nck binds to TCR initially through its SH3.1 domain and this binding recruits Src kinase through the other Nck SH3 domains to phosphorylate TCR and facilitate a secondary SH2-mediated Nck binding to tyrosine phosphorylated TCR (Gil et al., 2002; Lettau et al., 2010).

### Phosphorylation and Intramolecular Interactions

**FIGURE 2:**
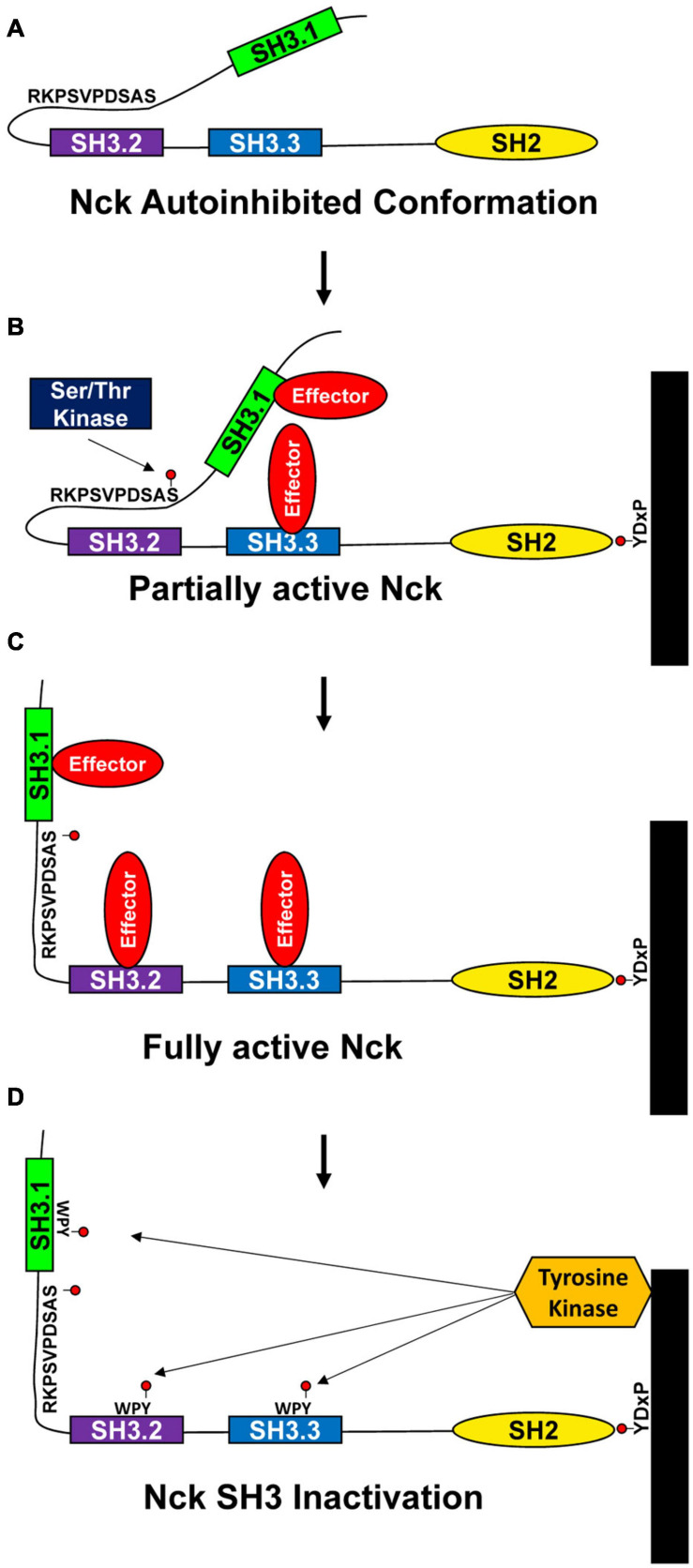
Proposed model for Nck1/2 regulation. **(A)** Intramolecular interactions between the Nck1/2 SH3.2 domain and a peptide in the linker between the SH3.1 and SH3.2 domains may limit SH3.2 interactions with other targets. **(B,C)** Phosphorylation of Ser85 in response to cell stimulation may reduce this intramolecular interaction to activate Nck1/2, allowing recruitment of effector proteins. **(D)** Tyrosine phosphorylation within the WPY motifs in the SH3 domains may limit SH3 domain interactions to inactivate Nck1/2.

While Nck1/2 undergoes inducible interaction with phosphotyrosine motifs and phosphorylation-inhibitable interactions with proline-rich motifs, another mode of Nck regulation may involve regulation of intramolecular interactions. Intramolecular interactions constrain the activity of several known kinases (e.g., Src, Janus, ZAP70), but less is known about intramolecular interactions regulating adaptor protein function (Kurochkina and Guha, 2013). Takeuchi and colleagues demonstrated a self-inhibitory intramolecular interaction between the Nck SH3.2 domain and a K/RxK/RRxxS sequence in the linker region between the SH3.1 and SH3.2 domain (Takeuchi et al., 2010). This interaction masks the Nck SH3.2 domain and may serve as a mechanism to limit Nck protein complex formation in the absence of stimulus (Figure 2A). Since this work used purified Nck protein, the relevance of the autoinhibited conformation to Nck function within the cell remains to be determined. Interestingly, the linker region between the Nck SH3.1 and SH3.2 domains is a site of extensive phosphorylation. While Nck1/2 undergoes phosphorylation in a variety of conditions (Li et al., 1992; Park and Rhee, 1992; Schmitz et al., 1997; Dionne et al., 2018), the function of these phosphorylation events remain virtually unknown. However, the inclusion of the Ser residue in this intramolecular inhibitory sequence (Ser85) may allow for phosphorylation-dependent relief of autoinhibition (Figures 2B,C). Multiple groups have shown Nck phosphorylation on Ser85 is associated with cell stimulation (Dephoure et al., 2008; Mayya et al., 2009; Konakahara et al., 2012; Dionne et al., 2018). Shear stress, H_2_O_2_, and hypoxia/reoxygenation injury all induce PAK2-Nck1/2 interactions, consistent with this interaction being inhibited under unstimulated conditions (Yurdagul Chen et al., 2013; Chen et al., 2015). In addition, phosphorylation of the conserved WPY triad in the Nck SH3 domains inhibits SH3 interactions as a negative feedback mechanism (Figure 2D) (Dionne et al., 2018). Proteomic analysis of liver ischemia-reperfusion injury identified Nck1 as a prominent tyrosine-phosphorylated protein (Emadali et al., 2007), suggesting that Nck tyrosine phosphorylation may be relevant to human disease. In addition to SH3 inhibition, Nck tyrosine phosphorylation sites may also induce intramolecular interactions with the Nck SH2 domain to inhibit SH2 domain function or may serve as a binding site for other SH2 and PTB domain containing proteins to enlarge the signaling complex.

## Major Functions of Nck 1/2

The Nck SH2/SH3 domains couple sites of tyrosine phosphorylation to the activation of downstream signaling partners that regulate cytoskeletal remodeling, gene transcription, protein translation, and cell survival (Li et al., 1992; Pawson and Scott, 1997; McCarty, 1998; Pawson and Nash, 2003; Pesti et al., 2012; Oeste and Alarcon, 2015; Porter and Day, 2016). However, depending on the extracellular stimulus and cell type, Nck1 and Nck2 may be capable of selectively regulating different responses within the cell (McCarty, 1998). In this section, we discuss the known role for Nck1/2 signaling in the regulation of cell function, and we identify potential roles for isoform-specific functions of Nck1 and Nck2 in this regard.

### Cytoskeletal Remodeling and Cell Migration

**FIGURE 3:**
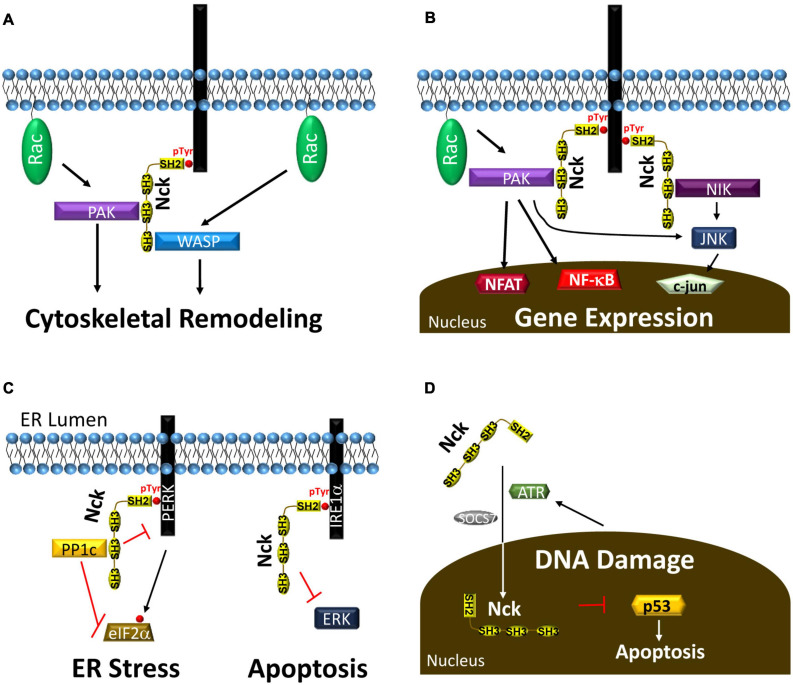
**(A)** Nck recruitment to tyrosine phosphorylated proteins recruits PAK and WASP to enhance their activation by the small GTPase RAC, leading to cytoskeletal remodeling and cell migration. **(B)** Nck recruitment to phosphotyrosine enhances activation of several proteins, such as PAK and NIK, that activate transcription factors to affect gene expression. **(C)** Nck1 recruitment to the ER membrane through interactions with PERK and IRE1α reduces ER stress and ER stress-associated apoptosis. **(D)** ATR and SOCS7 drive Nck translocation into the nucleus following DNA damage to reduce p53-dependent apoptosis.

Several previous reviews describe the important role for Nck1/2 in the regulation of cytoskeletal remodeling and cell migration (Li et al., 2001; Chaki and Rivera, 2013). Both Nck1 and Nck2 bind to hubs of tyrosine phosphorylation, such as pro-migratory activated growth factor receptors, chemorepulsive guidance molecule receptors, and sites of cell-matrix adhesions (Li et al., 1992; Meisenhelder and Hunter, 1992). After targeting these sites, Nck1/2 recruits and regulates several proteins involved in actin remodeling and cell migration (Li et al., 1992; Meisenhelder and Hunter, 1992). Nck1/2 recruitment to tyrosine phosphorylated receptor tyrosine kinases in the leading edge of migrating cells recruits the WASP/WAVE proteins to enhance Arp2/3 complex activation and actin branching (Figure 3A; Li et al., 2001; Chaki and Rivera, 2013). PAK recruitment and activation promotes turnover of actin filaments and integrin adhesions to enhance actin dynamics at the leading edge (Zhao et al., 2000; Stoletov et al., 2001). As such, Nck deficient cells fail to induce cytoskeletal remodeling and membrane ruffles in response to growth factor stimulation (Rivera et al., 2006). In addition, Nck1/2 recruitment to the leading edge regulates polarized cell migration by mediating activation of the polarity complex (cdc42, atypical PKC), suggesting roles in both actin dynamics and directed cell migration (Chaki et al., 2013). As cells migrate, formation of cell matrix adhesions stabilizes protrusions, perpetuate actin remodeling signaling, and mediate the actomyosin propulsive force (Huttenlocher and Horwitz, 2011). However, large adhesion sites limit cell migration by hindering release of cell-matrix adhesions during tail retraction (Huttenlocher and Horwitz, 2011). Nck1/2 binds to focal adhesion kinase (FAK) to facilitate recruitment to cell matrix adhesions. Focal adhesion targeting enhances cell migration through PAK recruitment, recruitment of the PAK-associated RacGEF, β-Pix, and focal adhesion turnover (Zhao et al., 2000; Stoletov et al., 2001). While Nck1 and Nck2 are often ascribed redundant roles in cell migration, this is not always the case. Silencing of Nck2, but not Nck1, blunts nerve growth factor-induced neurite outgrowth, associated with diminished paxillin levels (Guan et al., 2007). In contrast, axon retraction in response to EphA3 activation requires Nck1 binding (Hu et al., 2009). Furthermore, although both Nck1 and Nck2 expression can drive podosome formation, Nck2 deletion showed a stronger reduction than Nck1 deletion in reducing podosome-associated matrix invasion (Chaki et al., 2019).

### Transcription Factor Activation

Nck recruitment to phosphotyrosine signaling hubs promotes the activation of several signaling pathways that regulate gene expression. Tyrosine kinases classically activate the mitogen-activated protein kinase (MAPK) pathways resulting in activation of the c-fos/c-jun transcription factor, activated protein-1 (AP-1). Activation of c-fos by the ERK/MAPK pathway classically involves the SH2/SH3 adaptor protein Grb2, which recruits the RasGEF Son of Sevenless (SOS) to drive Ras-dependent ERK signaling (Buday and Downward, 1993; Egan et al., 1993; Gale et al., 1993). While early studies with Nck1/2 show SOS binding and Nck-dependent c-fos activation (Hu et al., 1995; Okada and Pessin, 1996; Gupta and Mayer, 1998), Nck1/2 does not contribute to ERK activation in response to several growth factors (Tanaka et al., 1995; Roche et al., 1996; Rockow et al., 1996). However, Nck1/2 regulates the c-jun N-terminal kinase (JNK) pathway by multiple mechanisms. Nck1/2 recruits Nck-Interacting Kinase (NIK; mitogen activated protein 4 kinase 4, MAP4K4) driving NIK-dependent JNK activation (Figure 3B; Su et al., 1997; Becker et al., 2000; Machida et al., 2004). In addition, Nck1/2 can also promote JNK through activation of Traf2- and Nck-interacting kinase (TNIK) or PAK (Schmitz et al., 2001; Poitras et al., 2003; Hahn et al., 2009; Shitashige et al., 2010). In contrast to the MAPK cascades, Nck1 shows a preferential activation of the proinflammatory transcription factors nuclear factor κ-light-chain-enhancer of activated B cells (NF-κB) and nuclear factor of activated T cells (NFAT; Figure 3B). Nck1, but not Nck2, mediates endothelial NF-κB activation in response to shear stress (Alfaidi et al., 2020a), and Nck1 recruitment to the T cell receptor drives PAK1-dependent NFAT activation (Yablonski et al., 1998; Ngoenkam et al., 2014). Finally, Nck1/2 binds phosphorylated nephrin in podocytes and sequesters/inactivates Lats1, thereby enhancing activation of the mechanically regulated transcription factor Yap, a key regulator of cell growth and proliferation (Keyvani Chahi et al., 2016).

### Protein Translation and Endoplasmic Reticulum Stress

In addition to regulating mRNA transcription, Nck adaptor proteins also regulate mRNA translation. Insulin stimulation promotes Nck1 recruitment into the enhanced ribosomal fraction through interactions between the Nck1 SH3.1/SH3.3 domains and eukaryotic initiation factor eIF2β (Kebache et al., 2002). Additionally, Nck1 upregulation enhances protein translation, suggesting that this targeting to the enhanced ribosomal fraction may promote translation (Kebache et al., 2002).

Nck signaling also regulates multiple aspects of the unfolded protein response (UPR) during endoplasmic reticulum (ER) stress. When misfolded proteins accumulate in the ER, UPR activation transiently slows translation and enhances expression of ER chaperones to facilitate cell recovery. The UPR consists of three distinct ER resident transmembrane stress sensors: protein kinase R-like ER kinase (PERK), inositol-requiring enzyme 1 alpha (IRE1a), and activating transcription factor 6 (ATF6) (Welihinda et al., 1999; Harding et al., 2000; Zhang et al., 2002). PERK phosphorylation stimulates eIF2α phosphorylation and inhibition of eIF2α-dependent mRNA translation (Cao and Kaufman, 2012). However, select genes, such as ATF4, show enhanced expression following eIF2α phosphorylation, allowing ATF4 to drive the expression of the antioxidant response, ER chaperones, and the apoptosis-associated protein CHOP. PERK phosphorylation on Y561 (pY^561^DxVxxD/E) recruits Nck1 to limit PERK activation and eIF2α phosphorylation (Figure 3C; Yamani et al., 2014). In addition, Nck recruits the phosphatase PP1c into this complex to dephosphorylate eIF2α (Latreille and Larose, 2006). IRE1α activation promotes the stabilizing splicing of the Xbp1 transcription factor, thereby allowing it to translocate to the nucleus and drive expression of ER chaperones and ER biogenesis genes (Cao and Kaufman, 2012). IRE1α phosphorylation promotes activation of JNK and NF-κB to drive proinflammatory gene expression (Cao and Kaufman, 2012). Nck interacts with proline-rich regions in IRE1α under normal conditions and represses ERK activation (Figure 3C). Activation of IRE1α in response to ER stress causes Nck1 release and ERK activation to promote cell survival (Nguyen et al., 2004). Therefore, Nck1 interactions with IRE1α and PERK serve to limit UPR signaling, and loss of Nck1 enhances cell survival following ER stress (Yamani et al., 2014, 2015).

### Cell Proliferation and Cell Survival

Nck1 and Nck2 show elevated expression in multiple cancers associated with enhanced proliferation and reduced apoptosis (Li et al., 1992; Labelle-Cote et al., 2011; Zhang et al., 2017). A five-fold overexpression of Nck drives the formation of an oncogenic foci (Li et al., 1992). In T cells, inhibiting the interaction between the Nck1 SH3.1 domain and the TCR with a small molecule inhibitor (AX-024) significantly reduces T cell proliferation both *in vitro* and *in vivo* (Borroto et al., 2014; Richter et al., 2020). While few studies have addressed the mechanisms by which Nck regulates cell proliferation, JNK inhibition reduces cell cycle progression in a variety of cell types (Himes et al., 2006; Wagner and Nebreda, 2009).

Nck expression regulates apoptosis through multiple mechanisms. Nck accumulates in the nucleus following DNA damage through an ATR- and suppressor of cytokine signaling 7 (SOCS7)-dependent mechanism, and Nck-depleted cells show enhanced apoptosis in response to DNA damage (Figure 3D; Errington and Macara, 2013). Depletion of either Nck1 or Nck2 enhances DNA damage-associated p53 activation and early apoptosis; however, Nck2 depletion had a significantly stronger effect (Errington and Macara, 2013). In contrast, Nck signaling can also promote apoptosis. Nck-dependent JNK activation promotes B cell apoptosis following B cell receptor activation (Mizuno et al., 2002), and Nck signaling is required for ER stress-induced CHOP expression and apoptosis (Li et al., 2013).

## Nck 1/2 in Vascular Biology

### Vascular Development

Nck1 and Nck2 are functionally redundant during development, as neither loss of Nck1 nor Nck2 expression in mice show any phenotypic changes (Bladt et al., 2003). However, deleting both Nck1 and Nck2 *in utero* results in embryonic lethality with defective chorion-allantoic fusion and axial rotation (Bladt et al., 2003). The Drosophila Nck homolog, Dock, regulates actin dynamics to facilitate neuronal growth cone guidance (Clemens et al., 1996; Rao and Zipursky, 1998; Li et al., 2001) and synapse formation (Desai et al., 1999). Similarly, neuronal-specific deletion of both Nck1 and Nck2 in mice results in deficient organization of the neuronal circuits critical for walking (Fawcett et al., 2007), such that neuronal Nck1/2 deficient mice display a hopping gate. Nck1/2 signaling within the developing spinal cord is required for outgrowth and growth cone architecture (Fawcett et al., 2007; Lane et al., 2015), and Nck has been identified as an essential downstream signaling component for EphB2-regulated axon guidance/growth cone collapse in rat cortical neurons (Srivastava et al., 2013). Regarding the vascular system, endothelial-specific deletion of both Nck1 and Nck2 during development results in embryonic lethality between E10 and E11 due to impaired angiogenesis, with reduced vessel branching and pericyte coverage (Clouthier et al., 2015). Endothelial cells from Nck1/2 knockout mice show significantly reduced migratory capacity, delayed cell spreading, and poor turnover of cell-matrix adhesions (Clouthier et al., 2015), consistent with a defect in angiogenesis. Interestingly, the endothelial-specific Nck1/2 deficient mice show defects in myocardial trabeculation and endocardial cushion formation (Clouthier et al., 2015). Endothelial-to-mesenchymal transition (EndMT), a phenotypic conversion involving reduced expression of endothelial marker genes (e.g., VE-cadherin) and enhanced expression of mesenchymal markers (e.g., smooth muscle actin, fibroblast-specific protein 1, fibronectin) (Chen P. Y. et al., 2020), drives endocardial cushion formation (Camenisch et al., 2002). Endothelial Nck1/2 deletion enhanced endothelial marker expression and reduced EndMT marker expression, suggesting that endothelial Nck1/2 signaling may affect endocardial cushion formation through EndMT regulation (Clouthier et al., 2015). In addition to fibronectin expression, fibronectin fibrillogenesis during EndMT requires contractility-dependent formation of elongated α5β1-rich fibrillar adhesions (Georgiadou and Ivaska, 2017). Consistent with Nck1/2-dependent fibronectin deposition in EndMT, endothelial Nck1/2 deletion reduces both adhesion length and α5β1-fibronectin adhesion force (Chaki et al., 2013).

### Postnatal Angiogenesis

In postnatal tissue, angiogenesis contributes to both physiological and pathological effects. In wound healing and tissue ischemia, angiogenesis enhances blood flow to promote tissue healing and homeostasis. However, in diabetic retinopathy, age-related macular degeneration, arthritis, and cancer, angiogenesis contributes to disease pathogenesis (Herbert and Stainier, 2011; Potente et al., 2011). In sprouting angiogenesis, quiescent endothelial cells respond to angiogenic factors by loosening cell-cell junctions and enhancing the production of matrix-degrading enzymes to allow for invasion of the adjacent tissue (Herbert and Stainier, 2011; Potente et al., 2011). Classic angiogenic factors, such as vascular endothelial growth factor (VEGF) and platelet-derived growth factor-B (PDGF-B), induce vascular cell migration and proliferation to promote new blood vessel formation (Lindblom et al., 2003; Koch and Claesson-Welsh, 2012).

#### Endothelial Nck1 and Nck2 in Angiogenesis

Tissue ischemia and inflammation drive local VEGF production, enhancing endothelial migration and sprout formation through the endothelial VEGF receptor 2 (VEGFR2). Although several studies have shown Nck recruitment to VEGFR2 (Stoletov et al., 2004; Wang et al., 2007), the specific isoforms involved remain unknown. A membrane-permeable peptide corresponding to a Nck-binding sequence in PAK1 (KPPAPPMRNTSTM, PAK-Nck peptide) significantly reduces endothelial migration *in vitro* and angiogenesis *in vivo* (Kiosses et al., 2002). Mice deficient in both Nck1 and Nck2 show deficient front-rear endothelial polarity in sprouting vessels, associated with severe angiogenic defects in the postnatal retina (Dubrac et al., 2016). While endothelial-specific Nck2 deletion partially reduces branchpoints and vascular density, Nck1 deletion has no effect on angiogenesis during retinal postnatal angiogenesis (Dubrac et al., 2016). Similarly, Nck1/2 deletion, and to a lesser extent endothelial-specific Nck2 deletion, reduces pathological angiogenesis in the oxygen-induced retinopathy model, whereas Nck1 deletion has no or minimal effect (Dubrac et al., 2016). Endothelial cells lacking both Nck1 and Nck2 show reduced migration and PAK2 activation in response to VEGF and the guidance molecule Slit2 (Stoletov et al., 2001, 2004; Chaki and Rivera, 2013; Dubrac et al., 2016), associated with reduced PAK activation and diminished focal adhesion dynamics. Similarly, endothelial cells deficient for Nck1/2 show reduced endothelial lumen formation in 3D culture (Chaki et al., 2015), associated with the reduced activation of the polarity pathway (cdc42, atypical PKC). In addition to polarity and actin dynamics, endothelial invasion into surrounding tissue often requires matrix degradation through the formation of podosomes, actin-based protrusions rich in proteolytic enzymes. Increasing Nck1/2 expression in endothelial cells drives podosome formation, whereas Nck1/2 depletion reduces pososomes (Chaki et al., 2019). Deletion of Nck2, and to a lesser extent Nck1, reduces endothelial cell invasion of a matrigel matrix, whereas rescuing Nck2 expression enhances matrigel invasion and gelatin degradation (Chaki et al., 2019). Interestingly, focal adhesion kinase phosphorylation on Y397 recruits Nck2 through an SH2-dependent mechanism, suggesting that Nck2 may play a preferential role in mediating cytoskeletal remodeling signaling from the focal adhesion (Goicoechea et al., 2002).

#### Pericyte Nck1 and Nck2 in Angiogenesis

Following the sprouting process, tube formation results in the initiation of blood flow and in vessel stabilization through pericyte recruitment (Herbert and Stainier, 2011; Potente et al., 2011). Endothelial cell-derived PDGF-B enhances pericyte recruitment via the pericyte PDGF receptor-β (PDGFRβ), whereas endothelial-specific deletion of PDGF-B results in diminished angiogenesis associated with reduced pericyte coverage and impaired vessel stability (Lindblom et al., 2003; Dubrac et al., 2018). Although PDGFRβ-dependent Nck1/2 recruitment has not been explored in pericytes, fibroblast PDGFRβ activation recruits both Nck1 and Nck2, with Nck1 binding to pTyr751 and Nck2 binding to pTyr1009 (Nishimura et al., 1993; Guan et al., 2009). Compared to Nck1 deletion alone, pericyte-specific Nck1/2 knockout mice show reduced angiogenesis associated with reduced vessel sprouts, reduced branchpoints, and diminished pericyte coverage (Dubrac et al., 2018). However, the effect of pericyte Nck1 or Nck2 deletion alone remains unknown. Nck1/2 knockdown in pericytes reduces PDGF-B-induced wound healing, associated with diminished PAK activation (Dubrac et al., 2018). In fibroblasts, deletion of either Nck1 or Nck2 reduces PDGF-B-induced migration (Ruusala et al., 2008), whereas overexpression of Nck1 or Nck2 SH2 domain or the SH3.2 domain (dominant-negative inhibitors of domain interactions) similarly blunts cell motility (Guan et al., 2009). The requirement for the SH2 domain (PDGFRβ-binding) and the SH3.2 domain (PAK-binding) is consistent with the PAK-dependent mechanism for reduced migration in Nck1/2 deficient pericytes (Dubrac et al., 2018).

### Vascular Permeability

The vascular endothelium provides a selective barrier limiting the efflux of fluid and plasma proteins into the surrounding tissue. Excessive fluid accumulation drives organ dysfunction in a variety of pathologies, and the leak of plasma proteins into atherosclerosis-prone arterial regions precedes plaque formation (LaMack et al., 2005; Orr et al., 2005) and may contribute to plaque-associated inflammation (Orr et al., 2005; Popov and Simionescu, 2006; Finney et al., 2017). Endothelial barrier integrity is mediated by endothelial adherens junctions (VE-cadherin, PECAM-1) and the tight junctions (claudin-5, occludin, JAMs). While endothelial transcytosis mediates active transcellular permeability (e.g, LDL), turnover of cell-cell adhesions and elevated cytoskeletal tension facilitate passive leak of fluids and plasma proteins through the induction of paracellular pores (Mundi et al., 2018). Phosphorylation of VE-cadherin in adherens junctions loosens its association with catenins and promotes adherens junction disassembly (Gavard, 2014). Additionally, myosin-mediated contractility significantly promotes vascular permeability (Sun et al., 2011). Therefore, the formation of paracellular pores likely requires both phosphorylation-dependent loosening of cell-cell junctions and contractility-dependent tension on cell-cell junctions.

#### PAK-Nck Interaction in Vascular Permeability

**FIGURE 4:**
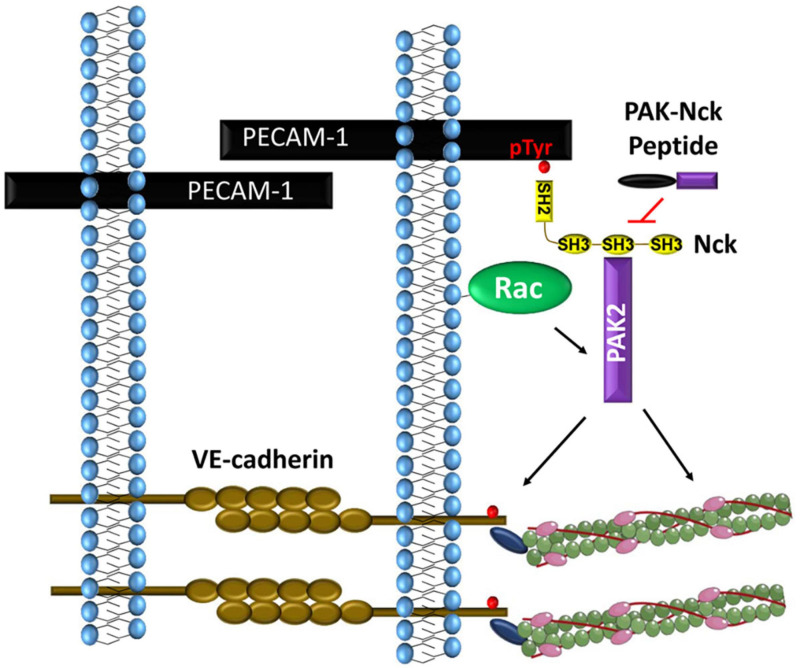
PECAM-1 phosphorylation recruits Nck1 and Nck2 to endothelial cell-cell junctions. Nck1-dependent PAK activation promotes VE-cadherin phosphorylation and local myosin-dependent contractility to induce paracellular pore formation.

Multiple lines of evidence suggest that Nck1/2 signaling critically regulates vascular permeability. Several permeability-inducing factors (VEGF, basic fibroblast growth factor, histamine, thrombin, and disturbed flow) promote PAK recruitment to endothelial cell-cell junctions (Figure 4; Stockton et al., 2004; Orr et al., 2007). Treatment with the PAK-Nck blocking peptide reduces PAK recruitment to cell-cell junctions, blunts myosin light chain phosphorylation, and limits paracellular pore formation in response to these factors (Stockton et al., 2004; Orr et al., 2007). In addition to PAK-dependent contractility, PAK-mediated VE-cadherin phosphorylation enhances both β-arrestin-mediated VE-cadherin endocytosis and endothelial permeability (Gavard and Gutkind, 2006). Furthermore, treating mice with the PAK-Nck peptide reduces vascular permeability in atherosclerosis, ischemia-reperfusion injury, and acute lung injury (Stockton et al., 2004; Orr et al., 2007; Reutershan et al., 2007; Chen et al., 2015). However, conflicting reports cloud the role of PAK in the permeability response. PAK activation promotes vascular integrity in several models (Garcia et al., 2001; Birukova et al., 2007), and deleting the major endothelial PAK isoform, PAK2, results in increased vascular permeability (Radu et al., 2015). Many of the studies implicating PAK as barrier protective suggest the lower expressed PAK1 isoform mediates these effects. However, these studies do not examine the relative roles of PAK1 and PAK2 in these processes, the specificity of the reagents for PAK1 is questionable, and the PAK1 knockout shows no permeability defects (Garcia et al., 2001; Birukova et al., 2007; Radu et al., 2015). One potential explanation for these discrepancies might involve the mechanism of PAK-recruitment to cell-cell junctions. Most studies showing PAK signaling enhances barrier function utilize signals that activate the cAMP/Protein Kinase A (PKA) pathway. PKA phosphorylates PAK2 within the Nck-binding sequence (KPPAPPVRMSST) and inhibits the PAK-Nck interaction, similar to the PAK-Nck blocking peptide (Howe and Juliano, 2000; Funk et al., 2010; Yurdagul Chen et al., 2013). However, PAK has additional proline-rich sequences that can bind Grb2 and β-Pix, and the interaction between PAK and β-Pix mediates the barrier-protective properties of PAK (Birukova et al., 2007). Therefore, PKA-mediated PAK2 phosphorylation may alter its function from barrier destabilizing to barrier protective, dependent upon changing which protein recruits PAK to the cell-cell junction.

#### Nck1 and Nck2 in Vascular Permeability

While these studies suggest a potential role for Nck in permeability, work from our laboratory has examined the mechanisms of Nck recruitment to the cell-cell junction and Nck isoform-specific signaling. Both shear stress and oxidant stress promote PECAM-1 tyrosine phosphorylation and the co-immunoprecipitation of Nck1/2 with PECAM-1 (Figure 4; Chen et al., 2015; Alfaidi et al., 2020b). PECAM-1 can be tyrosine phosphorylated at Y663 and Y686, and PECAM-1 lacking these sites fails to recruit Nck (Chen et al., 2015). While both pTyr sites (Y^663^TEV, Y^686^SEV) are similar to the Nck SH2-binding consensus sequence (Y-D/E-x-V/P) (Frese et al., 2006), isolated Nck1 SH2 and Nck2 SH2 domains show preferential interaction with the phospho-Tyr663 sequence (Machida et al., 2007). In response to shear stress, PECAM-1 phosphorylation recruits both Nck1 and Nck2. However, only deletion of Nck1 reduces shear stress-induced PAK2 activation and endothelial permeability, whereas Nck2 deletion paradoxically enhances both PAK activation and endothelial permeability (Alfaidi et al., 2020b). Domain-swap experiments combining the Nck1 SH2 domain with the Nck2 SH3 domains (and vice versa) suggest that the Nck1 and Nck2 SH2 domains play redundant roles in shear-induced endothelial permeability, whereas the Nck1 SH3 domains critically mediate this effect (Alfaidi et al., 2020b). This is consistent with the current model of PECAM-1 interaction with both Nck1 and Nck2, but only Nck1 mediating subsequent PAK activation. Since both Nck1 and Nck2 can promote PAK membrane translocation and activation (Bokoch, 2003), the reason for the preferential activation of PAK by Nck1 in this context is currently unknown. However, consistent with cell culture models, endothelial-specific Nck1 deletion reduces vascular permeability at sites of disturbed flow, whereas endothelial-specific Nck2 deletion does not (Alfaidi et al., 2020b). Since endothelial-specific Nck1 deletion does not result in a lethal phenotype similar to PAK2 deletion (Radu et al., 2015; Alfaidi et al., 2020b), Nck1 deletion may not limit all endothelial PAK2 signaling but rather the specific PAK2 signaling at the cell-cell junctions that promotes permeability.

### Proinflammatory Gene Expression

Endothelial activation, the conversion from a quiescent phenotype to a leaky, proinflammatory phenotype, contributes to a variety of cardiovascular diseases (Pober and Sessa, 2007). Proinflammatory cytokines (TNFα, IL-1β) and oxidized phospholipids promote endothelial activation, primarily through activation of the transcription factor NF-κB (Collins and Cybulsky, 2001). In atherosclerosis, disturbed blood flow patterns at vessel branchpoints, curvatures, and bifurcations induce cytoskeletal stiffening, elevated oxidant stress, and low levels of chronic NF-κB activation that primes these areas for further activation by atherogenic stimuli (Hajra et al., 2000; Hahn and Schwartz, 2009; Fang and Davies, 2012). Inhibiting NF-κB activation in endothelial cells protects mice from atherosclerotic plaque formation (Gareus et al., 2008), underscoring the importance of endothelial activation to atherosclerotic plaque formation. In contrast to disturbed flow, uniform laminar flow reduces endothelial activation and limits endothelial proinflammatory gene expression in response to circulating proatherogenic stimuli.

Laminar and disturbed flow induce distinct endothelial morphologies, which may contribute to their differential activation state. In response to laminar flow, transient activation of cytoskeletal remodeling pathways (e.g., integrin activation, focal adhesion kinase, Rac, PAK) promote both cytoskeletal remodeling in the direction of flow and activation of proinflammatory transcription factors, like NF-κB and YAP/TAZ, to induce proinflammatory gene expression (Hahn and Schwartz, 2009; Yurdagul and Orr, 2016). However, as cells align, these pathways are muted, and endothelial cells adopt a quiescent phenotype (Hahn and Schwartz, 2009; Yurdagul and Orr, 2016). In disturbed flow, sustained NF-κB activation occurs due to the inability of the cells to align, resulting in chronic activation of cytoskeletal remodeling signals (Hahn and Schwartz, 2009; Yurdagul and Orr, 2016).

#### PAK-Nck Interactions in Endothelial Proinflammatory Gene Expression

**FIGURE 5:**
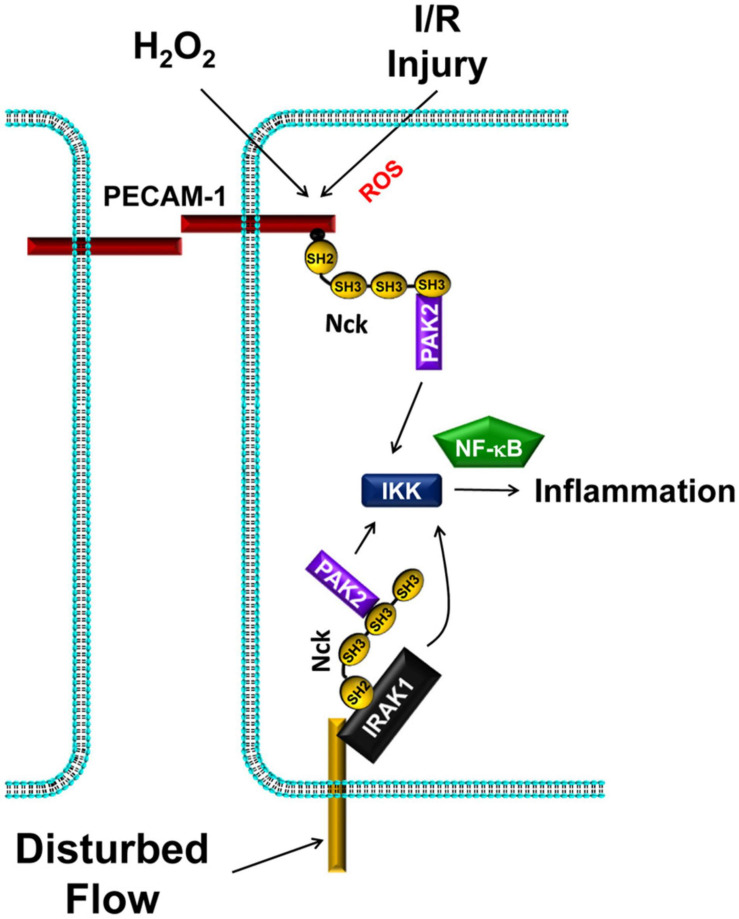
Nck1/2 recruitment to tyrosine phosphorylated proteins promote IKK-dependent NF-κB activation and proinflammatory gene expression. Oxidative stress (H2O2, ischemia/reperfusion injury) promotes Nck1/2 recruitment through PECAM-1 phosphorylation. In disturbed flow, Nck1 recruitment to IRAK-1 promotes NF-κB activation through a pathway that may or may not involve PAK2.

Early evidence for Nck’s role in proinflammatory endothelial activation derived from experiments utilizing the PAK-Nck peptide to inhibit PAK activation. Treating endothelial cells with the PAK-Nck peptide inhibited disturbed-flow-induced NF-κB activation, JNK activation, and proinflammatory gene expression (Figure 5; Orr et al., 2008; Hahn et al., 2009; Funk et al., 2010), and treating atherosclerosis-prone ApoE knockout mice with the PAK-Nck peptide prevented NF-κB activation, JNK activation, and proinflammatory gene expression at sites of disturbed flow *in vivo*. Mutation of the PKA-dependent phosphorylation site (Ser20) that inhibits the PAK-Nck interaction enhanced disturbed flow-induced NF-κB activation, suggesting that Nck-binding critically mediates this effect (Funk et al., 2010; Yurdagul Chen et al., 2013). Like disturbed flow, treatment with the PAK-Nck peptide significantly blunts the proinflammatory response to oxidative stress (Figure 5; Orr et al., 2008), and treating mice with the PAK-Nck peptide blunts inflammation in the cremaster ischemia-reperfusion injury model and the LPS-induced lung injury model (Reutershan et al., 2007; Chen et al., 2015). However, the selectivity of this peptide for Nck is questionable, as this peptide could target several other SH3 domain-containing proteins (Teyra et al., 2017). In addition, this peptide may target other non-endothelial cell types, as treating neutrophils with the PAK-Nck peptide significantly affects their cytoskeletal remodeling and cell migration (Reutershan et al., 2007).

#### Nck1 and Nck2 in Endothelial Proinflammatory Gene Expression

Work from our group sought to determine the direct role of Nck1/2 in vascular inflammation. Endothelial cells treated with siRNA targeting both Nck1 and Nck2 show reduced activation of NF-κB and the upstream IκB kinase (IKK) complex in response to exogenous (H_2_O_2_) and endogenous (hypoxia/reoxygenation) oxidative stress (Figure 5; Chen et al., 2015). Likewise, endothelial Nck1/2 depletion inhibits H_2_O_2_ and hypoxia/reoxygenation-induced expression of the proinflammatory adhesion molecules intercellular adhesion molecule-1 (ICAM-1) and vascular cell adhesion molecule-1 (VCAM-1; Chen et al., 2015). Similarly, Nck1/2 depletion prevents disturbed flow-induced NF-κB activation and ICAM-1/VCAM-1 expression (Alfaidi et al., 2020a). However, Nck1 and Nck2 play different roles in this regard. Nck1-depleted endothelial cells showed significantly less NF-κB activation and ICAM-1/VCAM-1 expression in response to disturbed flow, whereas Nck2 depleted endothelial cells do not (Alfaidi et al., 2020a). Furthermore, endothelial cells isolated from Nck1/2 double knockout mice fail to induce NF-κB activation and ICAM-1/VCAM-1 expression in response to disturbed flow, and re-expression of Nck1, but not Nck2, in these cells restores these proinflammatory responses (Alfaidi et al., 2020a). Moreover, atherosclerosis-prone ApoE knockout mice with a global Nck1 deletion show significantly reduced atherosclerotic plaque formation associated with diminished proinflammatory gene expression and leukocyte-positive plaque area (Alfaidi et al., 2020a). In contrast, endothelial-specific Nck2 knockout does not affect plaque formation or plaque-associated inflammation. While the effect of Nck1 deletion could be due to effects on leukocyte recruitment, bone marrow chimeras suggest that vessel wall Nck1 drives its proatherogenic effects (Alfaidi et al., 2020a).

Domain-swap experiments and point mutations in the Nck1 SH2 and SH3 domains suggest that specific Nck1 domains mediate this proinflammatory effect. Experiments with the Nck1/Nck2 chimeras suggest that the Nck1 SH2 domain critically mediates these proinflammatory signals, whereas the Nck2 SH3 domains can mediate the proinflammatory response when coupled with the Nck1 SH2 domain (Alfaidi et al., 2020a). Point mutations in the Nck1 SH2 targeting the critical Arg residue in the SH2 binding pocket (R308M) verify that disturbed flow-induced proinflammatory signaling requires the Nck1 SH2 domain (Alfaidi et al., 2020a). While the Nck1 and Nck2 SH3 domains appear to be redundant, inactivating point mutations in the Nck1 SH3 domains targeting the critical WPY triad (SH3.1 W38K, SH3.2 W143K, SH3.3 W229K) suggest that the Nck1 SH3.1 domain critically mediates flow-induced NF-κB activation and ICAM-1/VCAM-1 expression (Alfaidi et al., 2020a). This data is in conflict with the body of literature using the PAK-Nck peptide that should target the Nck SH3.2 domain to block interactions with PAK2. However, the SH3.1 domain provides a potential useful therapeutic target, as it is a non-canonical class I/II SH3 domain with an atypical PxLPxxDY binding sequence (Teyra et al., 2017).

While current data support a role for PAK in mediating endothelial activation, other Nck1-binding partners may also contribute to the inflammatory response. A study by Jacquet *et al.*, utilizing spatial proteomics to identify Nck1- and Nck2-selective binding partners, demonstrates a selective interaction between Nck1 and the interleukin-1 (IL-1) receptor (IL-1R) signaling partner IL-1R activated kinase-1 (IRAK-1) (Jacquet et al., 2018). IL-1 interaction with IL-1R initiates a signaling complex with MyD88 and IRAK-4 that recruits IRAK-1 to the plasma membrane, resulting in IRAK-1 phosphorylation on Thr209 (Abbate et al., 2020; Su et al., 2020). Active IRAK-1 binds TRAF6, and the resulting signaling complex becomes ubiquitinated with non-proteolytic K63-ubiquitin chains that recruit and facilitate IKK complex activation (Abbate et al., 2020; Su et al., 2020). Disturbed flow enhances endothelial Nck1-IRAK-1 interactions and Nck1-dependent IRAK-1 activation (Figure 5; Alfaidi et al., 2020a). While wildtype mice show enhanced endothelial IRAK-1 activation at sites of disturbed flow and in atherosclerotic plaques, Nck1 knockout mice show reduced IRAK-1 activation (Alfaidi et al., 2020a). Furthermore, IRAK-1 depletion significantly blunts disturbed flow-induced NF-κB activation and proinflammatory gene expression (Alfaidi et al., 2020a).

### Smooth Muscle Cell Function

Whereas several studies address the role of Nck1/2 signaling in endothelial cells and pericytes, only a few studies examine Nck1/2 signaling in vascular smooth muscle cells. Atherosclerosis-prone global Nck1 knockout mice show reduced smooth muscle cell incorporation into the plaque (Alfaidi et al., 2020a), which is typically driven by smooth muscle phenotypic modulation, migration, and proliferation. However, the role of Nck1/2 signaling in smooth muscle phenotype and smooth muscle migration has not been addressed. In fibroblasts, Nck1/2 deletion significantly reduces migration toward the classic smooth muscle cell mitogen PDGF-B, consistent with a potential role for Nck1/2 in mediating smooth muscle migration (Chen et al., 2000; Ruusala et al., 2008; Guan et al., 2009). In addition, multiple studies have shown a role for Nck1/2 in angiotensin-induced smooth muscle proliferation, as Nck1/2 depletion reduces angiotensin-induced smooth muscle proliferation associated with reduced activation of PAK and JNK (Voisin et al., 1999; Schmitz et al., 2001). Similarly, Nck1/2 depletion in fibroblasts blunts PDGF-B-induced proliferation (Roche et al., 1996). While these findings are suggestive, understanding how Nck1 and Nck2 signaling affects smooth muscle function will require additional studies.

## Current Clinical Therapies Targeting Nck Adaptor Proteins

The small molecule inhibitor AX-024 blocks the interaction between the Nck1 SH3.1 domain and the TCR and significantly reduces T cell proliferation both *in vitro* and *in vivo* (Borroto et al., 2014; Richter et al., 2020), suggesting that it may serve as a viable treatment for autoimmune diseases, such as psoriasis, autoimmune dermatitis, and multiple sclerosis (Borroto et al., 2016). This compound can be given orally, subcutaneously or intravenously and was well tolerated in mice even with higher doses. Phase Ia/Ib clinical trials have been completed in healthy human volunteers (Borroto et al., 2016; Flemming, 2017), but no efficacy data is currently available. In a mouse model of imiquimod-induced psoriasis, oral treatment with AX-024 significantly reduced skin thickening and inflammation. In ovalbumin-antigen-induced asthma model, AX-024 prevented airway sensitization and inflammation, and in an experimental model of autoimmune encephalopathy (a model of multiple sclerosis), treatment with AX-024 was more effective than the currently available treatment (Borroto et al., 2016; Flemming, 2017).

## Conclusion and Future Directions

The Nck family of SH2/SH3 adaptors proteins link activation of phosphotyrosine signaling hubs (activated growth factor receptors, sites of cell adhesion) to cytoskeletal dynamics and changes in gene expression driving cell proliferation, survival, and phenotypic changes. Nck1/2 signaling contributes to endothelial and pericyte cell migration to regulate vascular development and vascular remodeling in postnatal angiogenesis. In addition, Nck1/2 signaling contributes to the regulation of vascular phenotypic changes, such as endothelial activation and EndMT, through effects on gene transcription and protein translation. While several of these functions are redundant between Nck1 and Nck2, isoform selective effects have been described in multiple model systems, with Nck1 primarily implicated in vascular cell phenotype and Nck2 primarily implicated in vascular cytoskeletal remodeling and cell migration.

### Future Directions in Nck Research

Due to the similarity in domain structure and function, Nck1 and Nck2 have classically been viewed as redundant signaling mediators during organismal development. However, recent research highlights the isoform-selective roles of these proteins in various diseases, and new mouse models will be required to allow for selective inhibition of Nck1 and Nck2 in various cell types to fully characterize their context-specific functions. New advances in single cell RNA-Seq also promises to better enable our understanding of how Nck1 and Nck2 expression correlates with cell phenotype. In addition to expression, most binding partners show poor isoform selectivity, suggesting that new techniques may be required to characterize the Nck1-selective and Nck2-selective interacting partners under various conditions. Furthermore, the role of Nck1 and Nck2 post-translational modifications in the regulation of Nck1/2 function remain virtually unknown despite being known to occur for several decades. Future work will need to characterize how these modifications (primarily phosphorylation) affect Nck1/2 interactions and how they change during disease conditions.

Adaptor proteins provide a uniquely tunable therapeutic target. Redundancy within the receptor tyrosine kinases may limit the efficacy of therapeutics targeting individual enzymes. Therapeutics targeting kinase activity often result in significant negative side effects due to potent inhibition of target protein function and off-target effects on other kinases. In contrast, domain-selective inhibitors of the modular adaptor proteins block only a subset of their downstream effectors to limit specific signaling effects. For example, peptide inhibitors of the Nck1/2 SH3.2 domain reduce angiogenesis and show significant reductions in inflammation and permeability across multiple *in vivo* models. While this combination of effects may be useful for chronic inflammatory conditions like arthritis, the reduction in angiogenesis would likely limit their efficacy in atherosclerotic cardiovascular disease due to a worsened ability to respond to tissue ischemia. Since deficiency in either Nck1 or Nck2 does not disrupt angiogenesis, inhibitors targeting either Nck1 or Nck2 could affect isoform-specific effects without limiting angiogenesis or wound healing. Furthermore, inhibitors that target isoform-specific domains, such as the AX-024 small molecule inhibitor of the Nck1 SH3.1 domain, could further target specific downstream signaling effectors with limited off target effects. As current research focuses on high-throughput analysis of gene expression to identify potential therapeutic targets, analysis of protein-protein interactions and domain-selective protein functions will be necessary to utilize this information to treat human disease.

## Author Contributions

MA and AO wrote, edited, and generated the figures. MS wrote and edited the manuscript. All authors contributed to the article and approved the submission.

## Conflict of Interest

The authors declare that the research was conducted in the absence of any commercial or financial relationships that could be construed as a potential conflict of interest.
